# Ten simple rules to make the most out of your undergraduate research career

**DOI:** 10.1371/journal.pcbi.1005484

**Published:** 2017-05-04

**Authors:** Megan Yu, Yu-Min Kuo

**Affiliations:** 1 University of Virginia, Charlottesville, Virginia, United States of America; 2 Department of Cell Biology and Anatomy, College of Medicine, National Cheng Kung University, Tainan, Taiwan

In 2008, the Council on Undergraduate Research (CUR), a national organization founded in 1978 that promotes research opportunities for faculty members and undergraduates, featured 2,800 presenters in their annual undergraduate conference. Today, it has developed to include numerous disciplines ranging from biochemistry to theater and drama, and nearly 10,000 members and over 900 universities have participated in its endeavor to promote undergraduate research [1]. These statistics not only highlight the prevalence of undergraduates participating in research but also demonstrate the importance of research in undergraduate education.

Many undergraduates have reported numerous benefits from participating in research. In a study involving about 4,500 undergraduates that participated in undergraduate research opportunities sponsored by the National Science Foundation, respondents reported an increased level of understanding, resilience, and confidence in performing research and motivation to apply for graduate school programs [2]. In another analysis of 76 student interviews from four liberal arts colleges, undergraduates believed they have gained more laboratory (lab) techniques and have developed an attitude to “thinking and working like a scientist” [3]. These lab techniques and research attitudes are essential, as they help undergraduates develop better research habits and the solid foundation of knowledge and experience needed for their future research careers. For instance, knowing how to manage large datasets effectively, such as large patient genetic datasets and electronic health records, and designing proper algorithms and computational models to analyze data are essential skills for undergraduates interested in computational biology. In addition, unlike classroom learning, undergraduate research provides hands-on experience that allows undergraduates to gain a deeper understanding of the scientific process and to develop better research habits.

Despite the multiple benefits that research offers, undergraduates sometimes struggle and feel overwhelmed with the research process. Some undergraduates may not be familiar with the dynamics of the lab and may be afraid to interact with their lab colleagues and mentors. Other undergraduates may not completely understand the purpose of their work and feel overwhelmed by not knowing the results of their experiments before performing them. These consequences could, in turn, have detrimental effects on the relationship between undergraduates and their lab colleagues and decrease the motivation for undergraduates to pursue research in the future [4–5]. In light of these concerns, we propose ten simple rules constructed from our experiences as a college senior and a professor who has worked with undergraduate researchers that would help undergraduates enjoy and intellectually enrich their research experiences. Although this article may have components that are covered elsewhere [6–10], it extends and refines some advice from earlier articles so that they are more suitable for undergraduates.

## Rule 1: Start early

As an undergraduate, you may not know what type of research project you would like to pursue or whether it fits into your future research career. Therefore, it is essential to start early to explore and develop your research interests and goals. Your goal could be to gain more research experience before attending graduate school or to determine whether you prefer working in the industry to working in academia. Or, you might be new to research and hope to determine whether you would incorporate it into your future career or not. Whatever your reason is, be sure to start early to give yourself ample time to reflect on your goals and interests.

Finding the right research lab could take more than emailing several professors or research managers; it might require meeting a member of a lab at a conference or taking a tour of the lab to determine if it is the right fit. You might even consider joining professional research societies or research networks at your university to explore which areas are actively shaping the field and network with other researchers. For instance, the International Society for Computational Biology (ISCB) hosts numerous conferences and forums for computational biologists and students to network and promote their scientific research. It also has a career center for students and researchers to find jobs and be recognized for their talents [11]. Additionally, if you expect to publish during your undergraduate research career, you might want to start early to ask other professors and students within your department or look up the publication patterns of the potential mentor’s research group on the lab website.

As you become a new member of a lab, you might need some time to acclimate to the new lab environment and determine your commitment to doing research. You may find that life catches you off guard as you start to juggle between classes, jobs, and extracurricular activities, thus causing you to not find enough time to do research. Starting early, such as during your freshman or sophomore year, would provide you with ample time to explore your research goals and interests and participate in meaningful research activities.

## Rule 2: Know your foundational knowledge and skills

When you begin searching for undergraduate research positions, it is helpful to have already taken the recommended courses related to your research experience. Many professors would evaluate your knowledge and competence in a particular field to predict your success in the lab. Having the background knowledge in the research area of your chosen lab will help you understand the science behind the studies and experiments that are performed and will serve as useful foundational knowledge should you decide to pursue an independent research project in the future. For instance, while a computational biology lab might have a variety of lab members each with a different set of skills, such as a statistician, bioinformatician, or a software developer, it is helpful to have taken courses in computer science, programming, statistics, and biology before joining the lab. If your lab participates in a lot of programming activities, you might also consider brushing up on your coding and programming skills and taking a variety of Massive Open Online Courses (MOOCs) in computer science and programming [12–13]. Another way to gain more foundational knowledge is to read as much as you can about the topics pertaining to your chosen research lab from peer-reviewed journal articles, especially the papers that your chosen lab has published, or from popular science magazines. Table 1 lists some useful online resources for undergraduates to gain additional background preparation for their research experiences.

**Table 1:** Useful online resources for undergraduates in background research preparation.

Type of online resource	Relevant websites and URLs
MOOCs	Many companies, such as Udacity (https://www.udacity.com/), Coursera (https://www.coursera.org/), Khan Academy (https://www.khanacademy.org/), and edX (https://www.edx.org/), offer free online courses ranging from machine learning to business management. These courses may be helpful for undergraduates planning to do more interdisciplinary or specialized research.
Interactive coding and statistics websites	Codecademy (https://www.codecademy.com/), Code School (https://www.codeschool.com/), and Python for Biologists (http://pythonforbiologists.com/) offer free online training in programming and coding. Simple Interactive Statistical Analysis (SISA; http://www.quantitativeskills.com/sisa/), VassarStats (http://vassarstats.net/), OnlineStatBook (http://onlinestatbook.com/2/index.html), and Stat Trek (http://stattrek.com/) offer free online training in statistical analysis.
Scientific news and electronic databases	Many popular science magazines and websites, such as Scientific American (https://www.scientificamerican.com/), Discover (http://discovermagazine.com/), and Phys.org (https://phys.org/), cover the latest research findings in science and technology. Ovid (http://www.ovid.com/site/index.jsp), EMBASE (https://www.embase.com/), Web of Science (http://apps.webofknowledge.com/), and Google Scholar (https://scholar.google.com/) are popular electronic databases that allow you to search for many peer-reviewed articles and books.
Web forums and blogs	Many web forums, such as ResearchGate (https://www.researchgate.net/), allow you to ask questions and receive answers from experts when you start doing research. Biostars (https://www.biostars.org/) and SEQanswers (http://seqanswers.com/) are popular web forums in computational biology. Blogs, such as those from PLOS (http://blogs.plos.org/), The BMJ (http://blogs.bmj.com/), and Scientific American (https://blogs.scientificamerican.com/), are also popular online resources for learning.
International organizations	Many international organizations, such as the Global Organization for Bioinformatics Learning, Education & Training (GOBLET; http://mygoblet.org/training-portal) and the International Society for Pharmaceutical Engineering (ISPE; http://ispe.org/training), offer online training materials in highly specialized fields, such as engineering and computational biology.

While it is important to have the foundational knowledge before entering a lab, you should also remember to provide yourself enough time to do research (Rule 1). The process of finding the right time to do research can be complicated and may require you to seek additional help. For instance, you might consider discussing undergraduate research with potential mentors or advisers within your department. You might even visit the career center or take some research methodology or independent study courses at your university to determine if you are prepared. While starting research in your junior or senior year reduces the likelihood of publishing, you might have more foundational knowledge from your classes and have a more individualized approach to achieve your research goals and interests. Whatever it is you choose to do, make sure that you exploit the resources around you and give yourself enough time to decide when is the right time to do research.

## Rule 3: Let passion guide your research interests and goals

Like with many things in life, your interests and passions should help guide you to which research projects and fields you would like to pursue. Being interested in and passionate about the subject matter helps alleviate some of the mental and physical burden you may feel when spending countless hours in the lab. Before accepting an undergraduate research opportunity, you should ask yourself the following questions:

1. Is this research opportunity related to my academic interests?
2. What kind of research experience am I looking for, and what do I hope to gain from the experience?
3. How much time am I willing to commit, and what skills do I have that would contribute to this experience?
4. Do the professors whom I work for have similar academic interests as I do?

While many universities host many conferences and discussion forums in which professors, graduate students, and undergraduate researchers present their work, these events are also an opportunity for aspiring undergraduate researchers to meet with presenters and explore their academic interests. Take advantage of them! Exploring the websites of different research labs and other forms of apprenticeship should not be overlooked, as they are opportunities to gauge your interest in those fields and whether the research lab you are interested in is a good fit for you or not.

Moreover, being enthusiastic about the subject matter helps improve the chemistry you have with your lab director or with your research colleagues (Rules 4 and 6). While your colleagues and mentors are always willing to help you, it would make a better impression and would facilitate more dynamic discussions if you care about the topic. Your mentors and lab colleagues are also more motivated to help you with your project.

## Rule 4: Build positive relationships with your lab colleagues

As you become a new member of a research team, it is critical to be familiar with the dynamics of the lab and build good relationships with your research colleagues. Every lab has its own unique qualities. Some labs, such as basic science labs, may have a large team of senior researchers or graduate students performing experiments to investigate certain phenomena and developing assays on biofluid samples. Other labs, such as social science labs, may have a large team of graduate or undergraduate research assistants enrolling human participants to investigate a certain phenomenon. You might even have a research lab that involves a lot of collaborative research partnerships, sometimes international, with other labs. This is particularly true for labs that are largely interdisciplinary in nature or require highly technical equipment and expertise, such as a computational biology lab or a particle physics lab. There are also some labs that involve a small team of professors analyzing historical data, such as those in the humanities. Regardless of what type of research lab you are in, try to analyze the dynamics among the lab members, as this would help you acclimate to the new environment. You should also learn the expectations of your lab colleagues, as it would help you establish good research habits. Should you decide to have your own lab in the future, understanding lab dynamics and building good relationships with your research colleagues would help you understand your future undergraduate trainees and become a better mentor. Additionally, you should always treat your lab colleagues with respect as this would improve your relationship with them. They could serve as future collaborators, connections, or resources, as they may have more experience in certain research areas than you. Having occasional discussions or chats with them is another way to build better relationships with your lab colleagues.

## Rule 5: Keep an open mind and do not be afraid to ask questions

As an undergraduate, you may not be expected to know how to develop a research question that leads to a significant discovery and is feasible to answer within a limited amount of time. Or, you might be working in a large lab with so many open research questions and projects that you may not have the autonomy to develop your own research project. It is thus important to keep an open mind. Try to learn techniques and obtain new knowledge by having conversations with your senior colleagues. You should also allow your research mentor to guide you and give you advice, such as networking opportunities at professional research societies (Rule 1). Remember that learning how to do scientific research takes some time and effort (Rule 7), and your mentor is there to help you formulate your research project and guide you toward answering that question. Even after you have demonstrated competence in the lab, you should still keep an open mind, as there may be moments where you are inspired with a novel idea that may be relevant to your work. For instance, you might read an interesting news article about a study relevant to your research and wish to incorporate it into your project (Rule 10). Or, you might receive some useful advice from a conversation with a lab colleague and hope to include it into your work (Rule 4).

In addition to keeping an open mind, you should not be afraid to ask your senior colleagues any question regarding your research project or a particular research field. Asking questions is a great way to make an impression and foster open communication with your lab colleagues. It also allows you to learn more about a certain project you might not understand or any networking or presenting opportunity (Rule 9) that may be helpful for your future research career.

## Rule 6: Foster open communication with your research mentor and maintain a work/life balance

Research requires a significant amount of your time and energy and may take a mental and physical toll on your health, particularly if you are doing research during the school year. It is thus important to foster open communication with your research mentor. Remember that your mentor is providing you with the time and resources you need to succeed in the lab, so it is essential that you remain honest about your availability and work. Be sure to let your mentor know about your availability and goals working in the lab during the semester. Research should also be equally balanced with other extracurricular activities that you enjoy, as they would help you maintain a good work/life balance and could be helpful for your future career.

Fostering open communication with your mentor also demonstrates your initiative and progress to your mentor. As your research mentors may be busy with teaching and other scholarship endeavors, it is helpful to set up weekly meetings with them to demonstrate your progress and obtain constructive feedback for your work. You will build a stronger connection with your mentor and your mentor will be more likely to help you by writing you a strong letter of recommendation or helping you coauthor a peer-reviewed paper. If you encounter any moment in which the data you have collected do not meet your expectations, you should still discuss your progress with your mentor at least once per week, because you may fall into a vicious cycle in which you work hard to try to produce positive results in vain. During these instances, your mentor may slightly alter your research project so that you would not fall into that trap and lose motivation in doing research.

## Rule 7: Learn research by doing it

An important part of learning the scientific research process is to actually perform the research. Without setting up the experiment and testing your hypothesis properly, you will never know the truth about your research question [14]. As you perform the experiment, you might find an interesting discovery or gain more experience in doing a particular technique. Doing research also helps you develop better research skills and learn how to deal with setbacks. Regardless of what happens after you perform the experiment, try not to grow too attached to your data and do not put much stress on yourself if your study fails to produce significant results. Instead, you should remain confident and learn from your hardships. Be sure to also have open discussions about your results with other scientists or your lab group. While contributing to a peer-reviewed publication is definitely an impressive accomplishment that many undergraduates aspire to achieve, try not to give yourself too much pressure should your research contributions not turn out the way you expected or do not meet the standards required for a peer-reviewed publication.

Another helpful way to learn the scientific research process is to gain different or more diverse research experiences, especially when your interests change or if things do not turn out the way you have expected. These could be in the form of working on a different project in the same lab or transitioning to another lab to develop a different set of skills. For instance, if your goal is to become an experimental biologist to test for particular types of bioactivity of a drug or biomarker at the cellular or molecular level but your research experience thus far has only focused on data mining in large, biological databases, you might consider moving to a lab that focuses on developing high throughput assays that test these biomarkers and drugs. Whatever your choice is, be sure to let your mentor know of your decision and do the necessary background preparation you need to succeed in the next step of your undergraduate research career (Rule 2). You should also thank your former mentor and lab colleagues, as they have invested some time, effort, and resources in you.

## Rule 8: Be organized

Good organizational skills facilitate effective research and help you maintain a healthy lifestyle. Having an organized lab notebook or a folder with essential background research papers is critical for analyzing data or generating new ideas or proposals for your research project. Most importantly, being organized will help you tremendously when you present your results in a symposium or peer-reviewed publication, as it allows you to complete work in a timely manner. Good organizational skills also help you avoid being overwhelmed and overscheduling yourself with additional activities and other scholarly pursuits.

## Rule 9: Find opportunities to present your work

As an aspiring scientist, you should try to find opportunities to present your work. This could range from having an elevator pitch with a committee member to presenting your work at a conference or in a peer-reviewed paper. These opportunities would not only improve your communication and interpersonal skills and publicize your accomplishments but would also allow you to network with other scientists. Many universities host symposiums during the school semester, and some conferences allow undergraduates to submit an abstract for peer-review. In addition, many summer research programs and postbaccalaureate research programs host poster sessions or other conferences at the end of the research session for students to present their work.

## Rule 10: Keep up with the scientific literature

As an undergraduate researcher, it is helpful to keep up with the scientific literature, as it could provide some inspiration for your research project. For example, your research project might involve the role of a certain gene in the development of a disease and you might come across a scientific paper that cited a publicly available genetic database that could be helpful for your project. You might also have recently encountered an issue with your project for which another research group has just found a potential solution. These news items do not have to be peer-reviewed articles; they could include news from a variety of popular scientific news websites or magazines (Table 1). Keeping up with the scientific literature also helps you gain some additional background knowledge and skills you may need to use in your research project (Rule 2).

## Conclusion

Undergraduate research is an essential part of undergraduate education, as it offers many opportunities, ranging from developing the attitude to work as a researcher to networking and collaborating with other scientists. It is also fun and intellectually rewarding, as it allows you to uncover the truth about a phenomenon or develop better methods to investigate how the world works. These benefits are often not otherwise available in undergraduate education. Therefore, undergraduate research should be included in one’s undergraduate career if one is interested in pursuing research in the future. We hope that the advice and tips presented in this article will inspire and encourage other undergraduate researchers to enjoy and make the most out of their undergraduate research careers.
